# Temporary pacing following cardiac surgery – a reference guide for surgical teams

**DOI:** 10.1186/s13019-024-02619-9

**Published:** 2024-03-11

**Authors:** Steve W F R Waqanivavalagi

**Affiliations:** 1 Department of Medicine, University of Auckland, Auckland, New Zealand; 2 Cardiothoracic Surgical Unit at Waikato Hospital, Te Whatu Ora Waikato, Hamilton, New Zealand

**Keywords:** Temporary pacing, Cardiac surgery, Arrhythmia, Epicardial

## Abstract

Temporary pacing wires are often used following cardiac surgery to optimise the heart rhythm. Although setting and checking temporary pacemakers is typically undertaken by anaesthetists, intensivists, and nursing staff who care for post-cardiac surgical patients, almost all patients with temporary pacing wires are transferred to the ward with the pacing wires left in situ, where surgical, often junior, staff become responsible for temporary pacing wire management. Thus, knowledge is required not only of temporary pacing wire indications, types, and positioning at surgery, but also of practical skills in performing a pacing check, setting the pacemaker, and troubleshooting common problems. The available literature targets clinicians well-versed in temporary pacing wire management. However, this paper provides a practical ‘how to’ for surgical staff managing temporary pacing wires in a non-critical care environment.

## Introduction

Temporary pacing wires have been used since the 1960s to help manage cardiac rhythms in post cardiac surgical patients [1]. Arrhythmias remain common problems following cardiac surgery with up to 40% of patients experiencing atrial tachyarrhythmias [2]. Ventricular arrhythmias, and bradyarrhythmias such as sick sinus syndrome and atrioventricular blocks, are less common, but of greater clinical significance, with an increasing likelihood for permanent pacemaker placement. Setting and checking temporary pacemakers is typically undertaken by anaesthetists, intensivists, and nursing staff who care for post-cardiac surgical patients [3]. However, almost all patients with temporary pacing wires are transferred to the ward with the pacing wires left in situ, where surgical, often junior, staff become responsible for temporary pacing wire management. Thus, knowledge is required not only of temporary pacing wire indications, types, and positioning at surgery, but also of practical skills in performing a pacing check, setting the pacemaker, and troubleshooting common problems. The available literature targets clinicians well-versed in temporary pacing wire management. However, this paper provides a practical ‘how to’ for surgical staff managing temporary pacing wires in a non-critical care environment.

## Risk factors and indications

Increasing evidence suggests that the routine insertion of temporary pacing wires is unnecessary and that their application is required only in patients who are at high risk of pacing dependence [1]. In saying this, ongoing work is being undertaken to determine which patients fall into the high risk group. Thus far, a significant association has been made between the need for temporary pacing and risk factors including advancing age, valvular surgery, poor left ventricular function, structural heart disease, diabetes mellitus, preoperative beta-blocker or digoxin-use, and a history of arrhythmias [1–3]. 

## Types of pacing wires

Pacing wires use either unipolar or bipolar systems [1, 3, 4]. Unipolar pacing systems consist of a negative cathode and positive anode, which are respectively attached to the epicardium and subcutaneous tissue. Bipolar pacing systems consist of a single wire that possesses both an anode and cathode and is attached to the epicardium. In bipolar pacing systems, the shorter distance between the two poles means that the capture threshold is usually lower and the sensing threshold better, than unipolar pacing systems [4, 5]. Bipolar electrodes are also more suitable for dual chamber pacing because the risk of interference is reduced with the lower capture thresholds.

## Positioning of pacing wires

Temporary pacing wires are placed surgically towards the end of the case, prior to chest closure. Ventricular wires are sutured onto the diaphragmatic surface of the right ventricle, while the atrial wires are attached to the right atrium with sutures or clips [6]. Sutureless wires are a widely-used alternative to help simplify the pacing wire removal process. Some surgeons elect not to fix the electrodes to the heart but to instead maintain electrode contact by sandwhiching the electrode in place, such as between the heart and the diaphragm in the case of ventricular wire placement, or between the right atrial appendage and the aorta in the case of atrial wire placement. This latter method reduces the likelihood of causing cardiac tamponade from traumatic cardiac bleeding but is limited by the risk of temperamental contact and higher capture thresholds. The atrial wires are then brought out through the skin rightwards of the sternum while the ventricular wires are brought out through the skin leftwards of the sternum.

## Pacing check

A complete pacing check may be safely undertaken only if a patient is not pacing dependent, which might be the case if regular pacing spikes are seen on the rhythm strip. Slowly reducing the pacing rate until no pacing spikes are seen reveals both the underlying heart rate and rhythm. Caution should be exercised in patients who become haemodynamically unstable which, for patients in the ward whose blood pressure is not invasively monitored, may only manifest itself symptomatically. It is also not prudent to reduce the pacing rate below 30 bpm because doing so typically indicates that sinus node dysfunction or a high degree atrioventricular block exists, and electrophysiology review should be considered.

In patients who are not pacing dependent, a pacing check may commence with an assessment of the pacing wire sensing thresholds. *Sensitivity*, which is measured in millivolts, refers to the ability of the pacemaker to sense the current created by the underlying cardiac electrical activity. A pacemaker that is unable to sense the underlying rhythm is said to be *undersensing* and has a high sensitivity value. An undersensing pacemaker means that the pacemaker is unable to discern where in the cardiac rhythm a pacing spike should be delivered, which risks delivery of random pacing signals. For ventricular pacing wires, an undersensing pacemaker increases the chances of pacing on a T wave in a so-called R on T phenomenon, which can lead to ventricular fibrillation. Conversely, an *oversensing* pacemaker means that the pacemaker ‘over-reads’ the cardiac rhythm such that low voltage deflections are interpreted by the device as P waves or QRS complexes when they are not. In this setting, the opposite is true. The pacemaker will not discharge a pacing spike when in fact it should. Thus, pacing up will not occur for patients with an underlying heart rate below the set pacing rate. The minimum current that the pacemaker can sense is termed the *sensing threshold* and the sensitivity is often set to half this value to allow for detection of abnormally small signals and for the possibility that peri-lead fibrosis over the course of the day will reduce the current transmitted to the pacemaker. A common issue faced on the ward is of a low sensitivity threshold. It becomes unsafe to set the sensitivity of the device to half the sensing threshold, or even the lowest possible sensitivity setting, because of the risk of undersensing, and R-on-T.

The second concept required to be understood for setting the pacemaker is of the *output*, which is the current delivered by the pacemaker in milliamps. A low output means that the pacemaker is delivering a low current, while a high output means that the pacemaker is delivering a high current. The minimum current required to induce an actional potential in the myocardium is termed the *capture threshold* and can be observed practically as the minimum current required to produce regular pacing spikes with appropriate morphological rhythm changes. The output is often set to a value double that of the capture threshold. Thus, for a ventricular capture threshold of 2.5 mA, the ventricular output should be set to 5 mA. An inappropriately low output risks pacing signals having no, or indeed variable, effects on cardiac conduction. Whereas, an inappropriately high output risks extracardiac pacing such as of the phrenic nerves and acceleration of fibrosis at the lead/myocardium interface.

To check the ventricular sensing threshold, the atrial output should be switched off or the atrial pacing wire simply disconnected. The ventricular output should be set to the lowest possible value. The pacing rate should then be reduced to 20 bpm below the underlying heart rhythm. The ventricular sensitivity value should then be gradually increased from the minimum setting until the pacing device light-emitting diode (LED) illuminates regularly. The ventricular sensitivity value should then be gradually reduced until the sensing LED starts regularly illuminating. The sensitivity value at this point is the sensing threshold. The atrial sensitivity should then be assessed in a similar fashion. If a pacing wire sensitivity is too low, pacing modes dependent on that pacing wire should not be used. If neither of the pacing wires have adequate sensing, the pacemaker should be switched off.

To check the ventricular capture threshold, the ventricular sensing threshold must first be assessed as satisfactory. The pacing rate should be increased to 10 bpm above the underlying heart rhythm, with care taken in tachycardic patients. The ventricular output should then be gradually increased until regular pacing spikes are seen on the rhythm strip along with morphological changes. This value is the capture threshold, and the ventricular output should be set to a value double that of the capture threshold. In patients whose capture threshold approximates the maximal output setting, an early referral to electrophysiology should be considered, especially for patients who are pacing dependent, as an alternative pacing system may be required. The atrial capture threshold should be assessed in a similar fashion.

## Pacing modes

The pacing mode is classified in accordance with the Heart Rhythm Society and British Pacing and Electrophysiology Group generic code [4]. For single and dual chamber temporary pacing wires, a three-position code is used. Each position respectively codes the chamber paced, the chamber sensed, and the response to sensing. Ward-based pacing modes are usually either VVI, DDD, or AAI.

A VVI back up is the safest mode to use. It provides ventricular pacing when QRS complexes above the set pacing rate are not sensed and maintains adequate chronotropy if patients develop a high degree atrioventricular block or become bradycardic following rate or rhythm control-use.

DDD is a sequential pacing mode that senses both the atria and ventricles and paces either or both chambers if the set pacing rate is not sensed. In addition to ventricular pacing, DDD provides an atrial kick, which contributes an extra 25% to the cardiac output. DDD is termed sequential pacing because the atria and ventricles are paced independent of normal impulse conduction through the atrioventricular bundle. Thus, for patients who develop a high-grade atrioventricular block, they are not at risk of suffering from a low cardiac output state. In saying this, VVI and DDD pacing modes result in right ventricular pacing because ventricular pacing wires are routinely attached to the right ventricle.

Thus, utilising the native atrioventricular conduction system with AAI pacing in patients at low risk of atrioventricular conduction disease can increase the cardiac output because native left ventricular pacing is maintained. It is not possible to AAI pace patients who develop atrial arrhythmias as occurs commonly following cardiac surgery. Thus, unlike VVI and DDD pacing modes, AAI requires closer monitoring as atrial arrhythmia development can leave patients vulnerable to a lack of back-up pacing.

## Troubleshooting

Common problems with temporaring pacing include failure to pace, failure to capture, and R-on-T. Failure to pace occurs when the pacemaker is set to deliver electrical impulses but no corresponding pacing spikes are seen. There is a problem either with the pacemaker, the pacing wires, or at the lead/myocardium interface. A full check should be undertaken to evaluate whether the wires have migrated, whether the leads are properly inserted into the pacing box, and whether the pacing leads are faulty and require replacing. A pacing check should be undertaken to ensure that the pacing rate is not set below the patient’s intrinsic rate and that the device is not oversensing.

Failure to capture occurs when the pacemaker delivers electrical impulses that generate corresponding pacing spikes. However, the impulses do not reliably induce action potentials in the myocardium of the chamber being paced, which means that the patient’s heart rate remains lower than the rate set on the pacemaker. In this setting, a full check should be undertaken to eensure that there are no connection issues between the heart, pacing wires, pacing leads, and pacing box. A pacing check should be performed especially to confirm the capture threshold and appropriateness of the set capture output.

Inappropriate pacing, irregular pacing, or both, may occur in these circumstances: the set pacing rate is similar to the patient’s intrinsic rate, the capture output is set too low, or the device is oversensing or undersensing. If R-on-T occurs, the pacemaker should be switched off and senior input immediately sought. If the patient is pacing dependent, emergency help should be sought. It is important to check the sensing threshold and confirm both that the sensing threshold is not too low and that the sensitivity has been appropriately set.

## Removal of pacing wires

When patients no longer require temporary pacing and are evaluated as being at low risk of developing clinically significant conduction disease, the temporary pacing wires may be removed. In patients at low risk of thromboembolism, it may be prudent to with-hold prophylactic anticoagulation. In high risk patients, such as those with a mechanical mitral valve, a more acceptable balance of risk might be to remove the pacing wires with the anticoagulant at the lower end of the therapeutic range. Pacing wires should be removed prior to initiating direct oral anticoagulants [3]. The recommended upper limit for the INR in warfarinised patients varies widely but has been accepted up to 3.0 [7]. Thrombocytopaenia should also be allowed to self-correct until the platelet count is at least 100 E^9^/L. Temporary pacing wire removal is a sterile process that involves cleaning the area where the wires protrude through the skin with an anti-septic solution, cutting the sutures, and then applying gentle, but purposeful, traction towards the feet [1, 3]. Excessive force is not required. In fact, significant resistance may suggest that the pacing wire has either been retained with a stray suture, is firmly attached to the heart, or more commonly, has become lodged at the skin. In such cases, it may be wise to apply gentle traction as far as possible and then cut the wires at the skin, allowing for retraction of the wire internally during recoil [3]. Wire retention is not commonly problematic although serious complications have been reported.

## Complications

Although temporary pacing wire-use is considered safe, there is a small but appreciable risk of developing complications [1]. Perhaps the most concerning complications are tamponade, ventricular perforation, ventricular arrhythmias, and disruption of coronary anastomoses [1, 3]. Pacing wires are also at risk of localised infection and myocardial damage. More common issues pertain to their function. Over time, the capture thresholds increase while the sensing thresholds decrease. Epicardial oedema, inflammation, and fibrosis at the lead-myocardium interface are implicated in these changes, which affect the atrial wires more commonly than the ventricular wires [1, 8]. 

## Transition to permanent pacing

Following cardiac surgery, a small proportion of patients will require transition to permanent pacing [3]. Although the optimal timing is dependent on the clinical circumstances, it is reasonable to consider transition at the 4- to 5-day mark, although many centres do not place permanent pacing systems until 7 to 14 days, because most conduction problems improve with time [1, 3]. Common reasons for permanent pacing include high degree atrioventricular block (third degree and second degree with low ventricular rate) and sinus node dysfunction.

## Conclusion

In addition to understanding temporary pacing risk factors, indications, types, and positioning at surgery, it is also important for surgeons to know how to perform a pacing check, set the pacemaker, and troubleshoot common temporary pacing problems in post cardiac surgical patients, as outlined in this paper. A low sensing threshold risks R-on-T, while a high capture threshold risks pacing wire failure, which could have catastrophic effects in pacing dependent patients. For normally functioning pacing wires, a VVI is a safe back-up pacing mode. DDD is also safe, but preferable for use in patients who need sequential pacing due to known or high risk atrioventricular block. AAI preserves the native atrioventricular conduction system and is ideal when atrial kick and pacing up is required to increase blood pressure.

## Data Availability

Not applicable.
